# Non-tuberculous mycobacteria: occurrence in skin test cattle reactors from official tuberculosis-free herds

**DOI:** 10.3389/fvets.2024.1361788

**Published:** 2024-01-31

**Authors:** Alberto Gomez-Buendia, Julio Alvarez, Javier Bezos, Jorge Mourelo, Javier Amado, Jose Luis Saez, Lucia de Juan, Beatriz Romero

**Affiliations:** ^1^VISAVET Health Surveillance Centre, Universidad Complutense de Madrid, Madrid, Spain; ^2^Departamento de Sanidad Animal, Facultad de Veterinaria, Universidad Complutense de Madrid, Madrid, Spain; ^3^Servicio de Sanidad Animal, Xunta de Galicia, Consellería de Medio Rural, Santiago de Compostela, Spain; ^4^Servicio de Laboratorio de Sanidad Animal y Vegetal, Dirección General de Ganadería y Sanidad Agraria, Consejería de Medio Rural y Política Agraria, Principado de Asturias, Spain; ^5^Subdirección General de Sanidad e Higiene Animal y Trazabilidad, Dirección General de la Producción Agraria, Ministerio de Agricultura, Pesca y Alimentación, Madrid, Spain

**Keywords:** cattle, diagnosis, officially tuberculosis-free (OTF) herds, interference, non-tuberculous mycobacteria, tuberculosis, skin tests

## Abstract

Non-tuberculous mycobacteria (NTM) are considered a relevant cause of non-specific reactions to the most widely applied bovine tuberculosis (bTB) test, the intradermal tuberculin test. In order to establish which NTM species might act as a potential source of such diagnostic interference, a collection of 373 isolates obtained from skin test positive cows from 359 officially tuberculosis-free (OTF) herds, culled in the framework of the bTB eradication campaign in Spain, were identified at the species level through PCR and Sanger sequencing of the 16S rDNA, *hsp65* and *rpoB* genes. Of the 308 isolates for which a reliable identification was achieved, 32 different mycobacterial species were identified, with certain species being most represented: among *M. avium* complex members (*n* = 142, 46.1%), *M. avium* subsp. *hominissuis* (98; 69.0%) was the most abundant followed by *M. avium* subsp. *avium* (33, 23.2%), and *M. intracellulare* (7, 4.9%). Among non-MAC members (*n* = 166, 53.9%), *M. nonchromogenicum* (85; 27.6%) and *M. bourgelatii* (11; 5.6%) were the predominant species. In addition, mixed results were obtained in 53 isolates presenting up to 30 different genotypes, which could be indicative of new mycobacterial species. Our results represent a first step toward characterizing the diversity of NTM species that could interfere with official diagnostic tests for bTB eradication in Spain.

## 1 Introduction

The genus *Mycobacterium* is very large, encompassing 196 different child taxa with validly published and correct names described (1). Mycobacteria can take on different roles: there are highly relevant animal and human pathogens such as *M. bovis* and *M. caprae*, members of the *Mycobacterium tuberculosis* complex (MTC) and the most common causative agents of bovine tuberculosis (bTB), while others like the non-tuberculous mycobacteria (NTM) are typically free-living microorganisms widely distributed in the environment. These NTM can be found in soil, water, dust, etc. (2), but in certain cases, usually linked to immunosuppressive processes, they can infect humans and animals and act as opportunistic pathogens (3). In cattle, infection with NTM has not been traditionally associated with the presence of clinical signs [with the notable exception of *M*. *avium* subsp. *paratuberculosis* (*Map*), the causative agent of paratuberculosis], although the presence of granulomatous lesions as a consequence of infection has been described (4–7).

Since the beginning of the establishment of bTB eradication programs in different regions of the world in the early 1900s, several countries have been successful in the eradication of the disease (8). In others, however, despite all the efforts made, the disease is still endemic (9). Failure of these programmes has been partly attributed to the limitations in the sensitivity and specificity of existing diagnostic tests. Among factors compromising the performance of these tests, NTMs have been repeatedly linked to the occurrence of non-specific reactions in the single and comparative intradermal tuberculin tests (SIT and CIT), the main diagnostic tools used as the basis of eradication programs worldwide (10).

Most studies evaluating the role of NTM in bTB diagnostic problems have focused on those produced by *Map*, showing that it can affect both specificity and sensitivity (11–14). Nevertheless, NTM other than *Map*, also known as atypical or environmental, can also impact the reliability of bTB diagnostic tests due to the occurrence of cross-reactions in TB-free animals (15, 16), while their impact on the sensitivity of the tests remains to be quantified. Several studies have identified which species may be most commonly associated with these cross-reactions on bTB diagnostic tests in different settings, with *M*. *avium* subspecies and *M. nonchromogenicum* being the most commonly retrieved mycobacteria from bovine samples (15, 17–19). However, not all samples evaluated in these studies were from test positive animals or officially tuberculosis-free (OTF) farms. For instance, in Spain a recent study describing the presence of NTM in animals included only three reactor cattle from OTF herds, with other isolates originating from herds in which the disease was present, thus making the role of these bacterial species on the occurrence of cross-reactions unclear (19).

Therefore, the objective of this study was to identify the species associated with non-specific reactions detected in the framework of the bTB eradication campaign in Spain in a large cohort (*n* = 373) of skin test-reactor cattle from OTF herds located in areas with different prevalence of the disease. This information can be useful for better understanding the primary species affecting the specificity of skin tests for bTB and to assess the geographical variability in their occurrence.

## 2 Materials and methods

**Figure 1:**
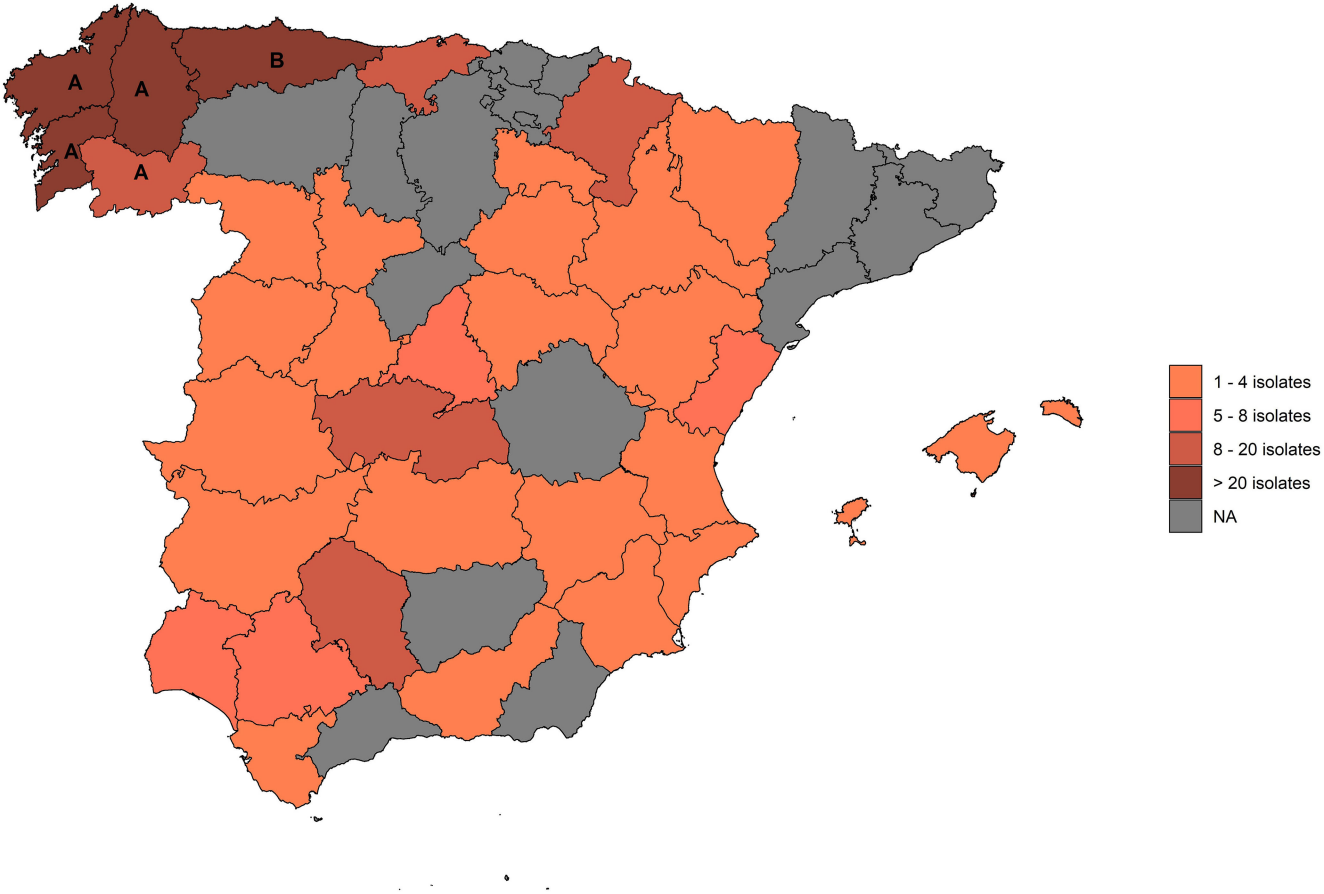
Provinces from which isolates included in this study originated. **(A)**: Galicia; **(B)**: Asturias.

All NTM isolates available at the VISAVET Health Surveillance Center retrieved between 2011 and 2020 from skin test-positive cattle located in 359 OTF herds detected in the frame of the Spanish eradication program were evaluated. Isolates were either cultured directly at VISAVET from cattle samples collected at the abattoir and analyzed in the laboratory there or submitted by official veterinary laboratories located in two OTF regions (Asturias and Galicia, 72.1% of the herds), with the remaining sampled animals originating from different parts of the country (Figure 1). Veterinary laboratories submitted the isolates to VISAVET for identification once they had confirmed they did not belong to the *M. tuberculosis* complex except for the OTF region of Asturias, which mostly submitted isolates already identified as belonging to the *M. avium* complex (MAC).

**Figure 2:**
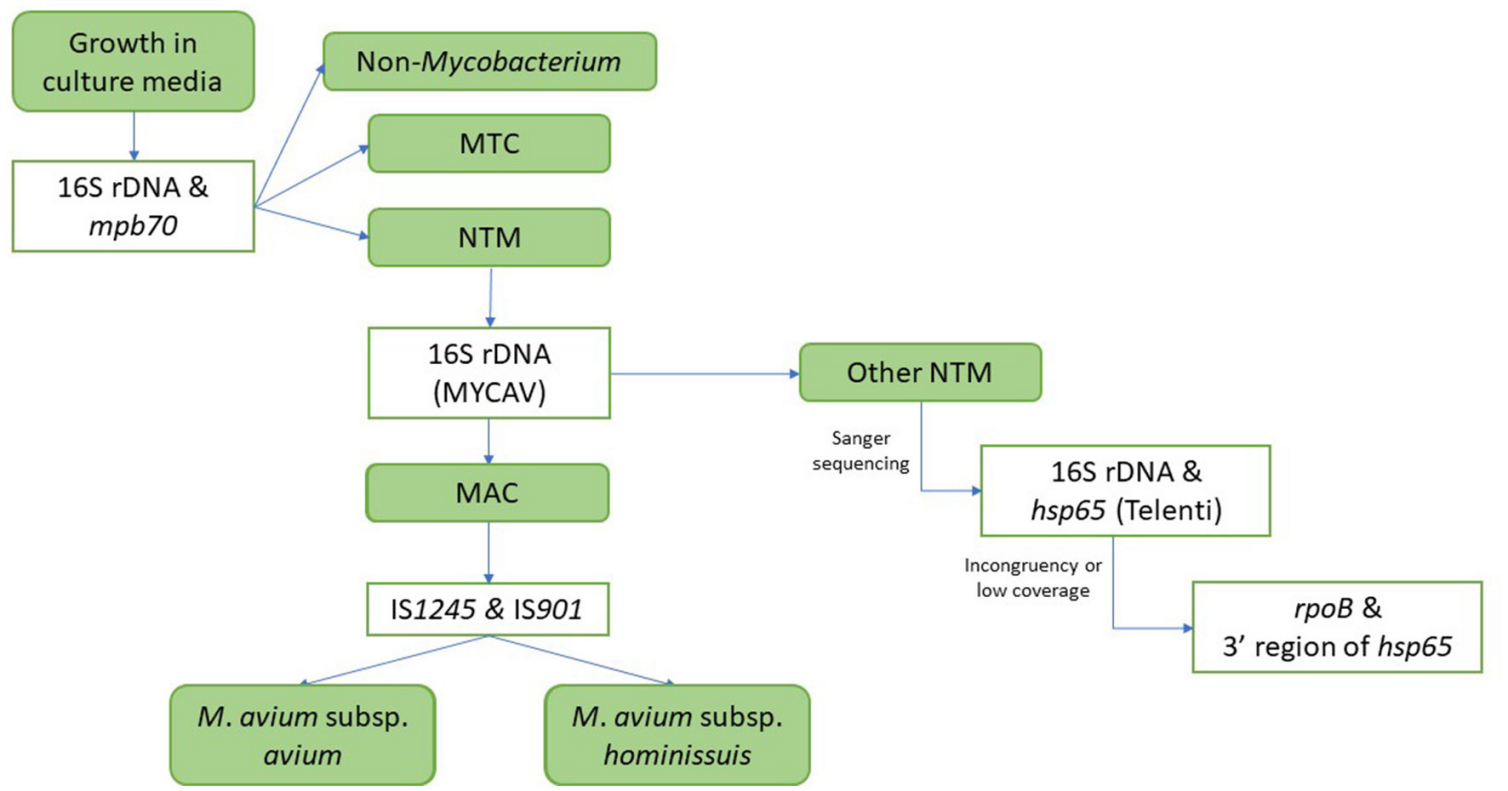
Workflow for the identification of isolates. MTC, *Mycobacterium tuberculosis* complex; NTM, non-tuberculous mycobacteria; MAC, *Mycobacterium avium* complex; MYCAV, MAC-specific 16S rDNA PCR.

According to the eradication program, when a skin test positive animal is identified in an OTF herd the animal must be culled within a maximum of 15 days and tissue samples are collected in the abattoir and submitted to an authorized laboratory in order to isolate or detect in the samples the presence of MTC members through bacteriology (20) and more recently direct PCR (21). Bacteriological analysis of these samples can occasionally lead to the identification of NTM species, which are identified as such in the laboratory according to the following protocol (Figure 2): once growth is observed, DNA is extracted and subjected to multiplex PCR for the detection of a *Mycobacterium*-specific DNA fragment (16S rDNA sequence) (22) and of a MTC-specific target (*mpb70* gene) (22). If the presence of a MTC member is not detected, another PCR aiming at a MAC-specific 16S rDNA (22) is performed, followed in case of a positive result by two PCRs aiming at the IS*901* and IS*1245* sequences associated with *M. avium* subsp. *avium* (*Maa*) (23) and *M. avium* subsp. *hominissuis* (*Mah*) (24). The available NTM strain collection included all isolates identified as *Mycobacterium* spp. based on the detection of the 16S rDNA *Mycobacterium* fragment but not belonging to MTC based on the absence of the *mpb70* gene that had been initially cultured at VISAVET or that were cultured in other authorized laboratories and later submitted to VISAVET for further identification. Isolates not belonging to MAC according to the methodology explained above were subjected to PCR amplification followed by Sanger sequencing of several targets: first, sequences of partial fragments of the 16S rDNA (22) and *hsp65* (Telenti fragment) genes (25, 26) were generated. Forward and reverse sequences were curated using BioEdit software (27) and combined to yield a consensus sequence that was then screened using the NCBI Basic Local Alignment Search Tool (BLAST) to identify the bacterial species. An identification was considered reliable if sequence similarity and coverage with a target was >99 and 100%, respectively (28). If the 16S rDNA/*hsp65* analysis yielded a non-reliable identification (due to low similarity/coverage and/or disagreement in the species identified through each target), amplification and sequencing of fragments of the *rpoB* gene (29) and then the 3′ region of the *hsp65* gene (30) was conducted and analyzed as described above. Full workflow is shown in Figure 2. Prior to bacteriological culture, samples were evaluated for the presence of macroscopial lesions compatible with bTB (31).

All primers used are shown in Supplementary Table 1. Sequencing was performed by STABvida (Lisbon, Portugal).

## 3 Results

Overall, 373 isolates recovered from positive cattle located in 359 OTF herds across 34 provinces (15 Autonomous Communities) were included in the study (Figure 1). Of these, 194 (54.0%) were beef herds, 150 (41.8%) were dairies, eight (2.2%) were fattening units, and seven (1.9%) were bullfighting herds. Of the 373 isolates, we were able to achieve a reliable identification in 308 (82.6%). Among these, 166 (53.9%) were classified as non-MAC NTM and 142 (46.1%) as MAC species. In the remaining 65 isolates (17.4% of all samples) a reliable identification was not achieved. Of them, 30 (46.2%) were classified as non-MAC NTM, 23 (35.4%) as MAC, and the 16S rDNA sequence revealed a bacteria not belonging to the *Mycobacterium* genus for the remaining 12 (18.5%) isolates.

Within the 166 non-MAC NTM isolates, 27 species were reliably identified (Table 1), being the most frequent species *M. nonchromogenicum* (*n* = 85; 51.2%), followed by *M. bourgelatii* (*n* = 11; 6.6%), *M. shimoidei* (8; 4.8%), *M. kansasii* (7; 4.2%) and *M. intermedium* (7; 4.2%). A reliable identification was not achieved in 30 isolates (15.3%), of which 8 and 16 did not yield a positive result for the *rpoB* and 3′ region of *hsp65* genes, respectively (Table 2). Among these isolates, we identified 23 “genotypes” present in 1–5 isolates. A genotype is defined as distinct partial 16S rDNA and *hsp65* (Telenti fragment), and, when available, *rpoB* and 3′ region of *hsp65* sequences. Most of the non-MAC NTM were retrieved from Galicia (141/196). However, the diversity of genotypes was almost the same as in the non-OTF regions (29 and 28 genotypes, respectively).

**Table 1:** NTM species with a reliable identification from cattle positive to skin test.

**NTM species**	**Isolates (%^a^)**
*M. avium* subsp. *hominissuis*	98 (31.8%)
*M. nonchromogenicum*	85 (27.6%)
*M. avium* subsp. *avium*	33 (10.7%)
*M. bourgelatii*	11 (3.6%)
*M. shimoidei*	8 (2.6%)
*M. intracellulare*	7 (2.3%)
*M. kansasii*	7 (2.3%)
*M. intermedium*	7 (2.3%)
*M. alsense*	5 (1.6%)
*M. parascrofulaceum*	5 (1.6%)
*M. smegmatis*	5 (1.6%)
*M. xenopi*	4 (1.3%)
*M. abscessus*	3 (10.7%)^b^
*M. palustre*	3
*M. colombiense*	2
*M. engbaekii*	2
*M. fortuitum*	2
*M. gilvum*	2
*M. holsaticum*	2
*M. interjectum*	2
*M. thermoresistibile*	2
*M. triplex*	2
*M. yongonense*	2
*M. aubagnense*	1
*M. bohemicum*	1
*M. chitae*	1
*M. frederiksbergense*	1
*M. heckeshornense*	1
*M. heraklionense*	1
*M. koreense*	1
*M. lentiflavum*	1
*M. paraense*	1

^a^Percent relative to the 308 isolates with identification.

^b^This represents the aggregated percentage of those species with less than four isolates.

**Table 2:** NTM isolates without a reliable identification from cattle positive to skin test.

**Genotype**	**Isolates**	**16S rDNA**	**% of similarity**	***hsp65* (Telenti fragment)**	**% of similarity**	***rpoB***	**% of similarity**	**3^′^region of *hsp65***	**% of similarity**
1	10	*M. colombiense/intracellulare/bouchedurhonense*	99.8	*M. scrofulaceum*	97.4	*Mycobacterium* sp.^a^	100	*M. timonense*	96.1
2	5	*M. marseillense/yongonense/intracellulare*	100	*M. avium/colombiense*	98.6	*M. mantenii*	96.2	–	–
3	5	*M. vaccae*	100	*M. vaccae*	93.4	–	–	–	–
4	2	*M. barrassiae/mengxianglii*	99.0	*Mycobacterium* sp.^b^	95.7	–	–	–	–
5	2	*M. chelonae/phlei*	99.8	*M. chelonae/phlei*	99.5	*M. chelonae/phlei*	99.6	–	–
6	2	*M. colombiense/intracellulare/bouchedurhonense*	100	*M. avium/colombiense*	98.5	*M. mantenii*	96.2	–	–
7	2	*M. colombiense/intracellulare/bouchedurhonense*	100	*M. avium/colombiense*	99.1	*M. vulneris*	99.9	–	–
8	2	*M. colombiense/intracellulare/bouchedurhonense*	100	*Mycobacterium* sp.^c^	99.8	–	–	–	–
9	2	*M. szulgai/angelicum*	99.7	*M. saskatchewanense/nebraskense*	95.9	*M. paraense*	92.0	*M. parmense*	94.2
10	1	*M. algericum/sinense/novum*	99.2	*M. algericum/terrae/sinense*	100	–	–	*M. novum*	97.5
11	1	*M. bacteremicum/neoaurum/sphagni*	100	–	–	–	–	–	–
12	1	*M. brasiliensis*	99.0	*M. komanii*	97.6	*Mycobacterium* sp.^d^	100	–	–
13	1	*M. colombiense/intracellulare/bouchedurhonense*	100	*Mycobacterium* sp.^e^	99.8	–	–	–	–
14	1	*M. engbaekii*	100	*M. arupense*	99.3	–	–	*M. virginiense*	96.8
15	1	*M. flavescens*	99.1	*M. monacense*	96.2	*M. baixiangningiae*	96.0	–	–
16	1	*M. interjectum/paraense*	100	*M. lentiflavum/genavense*	96.7	*M. interjectum/paraense*	97.6	–	–
17	1	*M. interjectum*	100	*M. saskatchewanense*	97.0	*M. interjectum*	98.8	–	–
				*M. yongonense/intracellulare/avium*					
18	1	*M. marseillense/yongonense/intracellulare*	100		99.8	–	–	–	–
19	1	*M. nonchromogenicum*	100	*M. icosiumassiliensis*	97.8	–	–	–	–
								*M. novum*/*sinense*	
20	1	*M. novum/sinense/algericum*	100	*M. senuense*	100	–	–		100
								*Mycobacterium* sp.^f^	
21	1	*M. scrofulaceum/paraffinicum*	100	*M. bohemicum*	96.6	*M. seoulense*	95.8		95.4
22	1	*M. septicum*	100	*Mycobacterium* sp. *^*g*^*	99.5	*Mycobacterium* sp.^h^	100	–	–
23	1	*M. terrae*	99.6	*M. parascrofulaceum*	100	*M. terrae*	94.6	–	–
								*Mycobacterium* sp.^f^	
24	1	*M. triplex*	99.0	*M. genavense*	97.2	–	–		95.4
25	1	*M. triplex*	99.7	*M. avium* subsp. hominissuis^*^	98.8	–	–	–	–
26	1	*M. triplex*	99.7	*M. parmense*	98.0	*Mycobacterium* sp.^i^	97.9	–	–
				*M. avium* subsp. *paratuberculosis*/*avium*/hominissuis^*^					
27	1	*M. triplex*	99.7		98.1	–	–	–	–
28	1	*M. vanbaalenii*	100	–	–	*M. aurum*	94.4	–	–
29	1	*M. vanbaalenii/vaccae*	99.7	–	–	*M. vaccae*	96.8	–	–
30	1	*Mycobacterium* sp.^j^	99.9	–	–	*M. heraklionense*	90.6	–	–

^a^Sequence ID: ON994929.

^b^Sequence ID: HF566126.

^c^Sequence ID: OK539021.

^d^Sequence ID: KM234049.

^e^Sequence ID: EU619890.

^f^Sequence ID: CP079863.

^g^Sequence ID: KT4550001.

^h^Sequence ID: OK538910.

^i^Sequence ID: ON994981.

^j^Sequence ID: MK890524.

^*^These samples were negative to MAC-specific 16S rDNA PCR (MYCAV).

For the 142 MAC isolates five species were identified (*Mah, Maa, M. intracellulare, M. yongonense*, and *M. colombiense*). Of these, *M. avium* subspecies accounted for 92.3% (*n* = 131) of the retrieved isolates, with *Mah* (98; 69.0%) being more represented than *Maa* (33; 23.2%). Most MAC isolates (63/165) originated from Asturias since this region submitted primarily cultures preliminarily identified as MAC, but 56 and 46 MAC isolates from Galicia and non-OTF regions, respectively, were also included. Regarding the predominant MAC species, *Mah* was recovered more frequently than *Maa* in all regions, and in the case of Galicia, *M. intracellulare* (4) was identified even more frequently than *Maa* (1).

Of the 23 isolates with non-reliable identification, we did not obtain a PCR amplicon for the *rpoB* and 3′ region of *hsp65* genes in 11 and 20 isolates, respectively. Among these 23 isolates, seven genotypes, as previously defined, were identified in between 1 and 10 isolates each. The most common genotype was found in 10 isolates from five provinces in which a 99.8% similarity with the 16S rDNA sequence of *M. colombiense, M. intracellulare*, and *M. bouchedurhonense* coupled with a 97.4% similarity with the *hsp65 M. scrofulaceum* gene sequence was obtained. All genotypes for isolates with a non-reliable identification are shown in Table 2.

Finally, the 16S rDNA sequence revealed that 12 isolates did not belong to the *Mycobacterium* genus but instead to the following species: *Prauserella rugosa* (*n* = 3), *Streptomyces hydrogenans* (3), uncultured actinobacteria (3), *Corynebacterium pseudotuberculosis* (2), and *Brevibacillus brevis* (1).

Overall, macroscopical granulomatous bTB-like lesions were observed in lymph node samples from 20 animals (5.3%). From these samples, 11 different species were identified: *Mah* (*n* = 6), genotype 2 (2) (Table 2), *M. nonchromogenicum* (2), *M. shimoidei* (2), *Maa* (1), *M. intracellulare* (1), *M. xenopi* (1), *Corynebacterium pseudotuberculosis* (1), *M. interjectum* (1), *M. yongonense*, and genotype 7 (1) (Table 2).

## 4 Discussion

Non-tuberculous mycobacteria are one of the limiting factors compromising diagnostic performance of bTB tests (15, 32). As the burden of bTB is decreasing in most countries in which eradication campaigns have been consistently applied, the need to maintain a high specificity is key to keeping the positive predictive value as high as possible, but without compromising the overall sensitivity of the surveillance system. The characterization of the factors affecting diagnostic specificity, such as the most prevalent NTM associated with non-specific reactions to bTB skin tests, can be useful to design strategies aiming at minimizing their impact. Here, we present a thorough characterization of a large panel of NTM isolates retrieved from skin test reactor cattle from OTF herds to assess their diversity in Spain.

A great percentage of the isolates included in the study belonged to MAC (specifically to the *Mah, Maa, M. intracellulare, M. colombiense*, and *M. yongonense* species, of which the first two were by far the most common), partly due to the fact that the second region from which a higher number of isolates were available (Asturias) provided mostly NTM preliminary identified as MAC (Table 1) (33). Nevertheless, the high frequency of isolation of MAC members from cattle here is in agreement with previous studies in which certain MAC members (*Mah, Maa, Map, M. arosiense, M. bouchedurhonense, M. chimaera, M. colombiense, M. intracellulare*, and *M. vulneris*) were also isolated from bovine samples and identified as a potential cause of non-specific reactions (15, 19, 34). The potential to elicit a cross-reacting immune response in bTB diagnostic tests by MAC members (and to some extent also by other NTMs) should be reduced by the use of the CIT, which also considers the response elicited by the avian protein purified derivative (PPD-A) obtained from a *Maa* isolate (35, 36), so diagnostic interference should be limited in areas where this test is used (33). This, however, can lead to a decrease in diagnostic sensitivity of between 14 and 44 percentage points according to some estimates (37), and therefore comparative tests should be used when the risk of tuberculosis is considered low. In our study, most of the animals (353/373) were reactors in the SIT (based only on the bovine PPD), and in fact, of the only 20 animals that were reactors in the CIT (all coming from a current OTF area), *M. nonchromogenicum* was isolated from 13 of them and only one MAC species (*M. colombiense*) was isolated, suggesting that the CIT could be compromised to a larger extent by certain mycobacterial species.

*M. nonchromogenicum* was the most frequently isolated non-MAC NTM. This species was the most common NTM retrieved in samples from cattle in Northern Ireland (23 of 48 animals) (18) and Hungary (30 of 104) (17), and the second most common in France (81 of 310) (15), although in these studies not all samples came from skin-positive animals or OTF herds. *M. nonchromogenicum* has also been described in cattle samples with lesions collected at abattoirs in Switzerland (6), South Africa (34) and Ethiopia (7, 38), as well as in milk samples from positive cows to the CIT in Brazil (39) and in the nasal mucosa of cattle (40). In another study carried out in Spain (19), including tissue samples from cattle and wild boar in the Basque country (from which no samples were included here), *M. nonchromogenicum* was isolated in three of 21 SIT positive cattle and nine wild boars. These results, coupled with our findings, demonstrate that *M. nonchromogenicum* can be found in reactor cattle from Northern regions in Spain, from which most isolates were retrieved, and could be a relevant contributor to the development of cross-reactions in the skin test. The ability of *M. nonchromogenicum* to elicit reactions after the inoculation of certain mycobacterial antigens (including PPD-A and PPD-B) was explored in an experimental infection model in guinea pigs, but the observed induced reactions were limited (16). Similarly, the ability of this species to cause false positive reactions in skin tests in cattle has not been demonstrated yet, since experimental studies to assess the cross-reaction potential of NTM have been only limited to *M. kansasii* (4, 41) and *M. fortuitum* (42).

Regarding the geographical distribution of non-MAC NTMs, 93% of the 85 *M. nonchromogenicum* isolates identified here were recovered from cattle from Galicia, representing 56% of all non-MAC NTMs available from this particular region. In contrast, no non-MAC NTM accounted for more than 10% of the isolates retrieved from non-OTF regions, suggesting a more heterogeneous distribution of NTM species. When considering MAC isolates, *Mah* was the most commonly found species, but its relative frequency compared with other MAC species varied between regions: in Galicia 85.4% of all MAC isolates with a reliable identification (41/48) were *Mah* and only one *Maa* isolate was retrieved. In contrast in Asturias 34 *Mah* and 25 *Maa* isolates were identified, accounting for 57.6 and 42.4% of the 59 MAC isolates with reliable identification, respectively, while in non-OTF regions *Mah* and *Maa* represented 65.7 and 20.0% of the 46 MAC isolates available from these regions, respectively. Although these differences may be pointing out at differences in the epidemiology of cross-reactions due to NTM in cattle between OTF and vs. non-OTF regions, the highly heterogeneous sampling efforts may have led to biases contributing to these results and thus these findings should be interpreted with care. Differences in the most prevalent NTM species associated with cross-reactions were not influenced by the production type, with *M. nonchromogenicum* being the most abundant in both beef and dairy herds in Galicia (42.3 and 38.4% of all isolates in each type, respectively). Similarly, the distribution of MAC members across different production type herds in Asturias was also similar, with *Mah* being isolated in 52.5 and 56.5% of the beef and dairy herds, respectively, and *Maa* in 40.0 and 34.8% of them. This suggests that the prevalence of the NTM may be more influenced by regional factors rather than linked to the type of production.

Interestingly, 53 isolates in our study could not be reliably assigned to a specific species. Among them, 10 isolates coming from beef herds exhibited identical sequences in the four sequenced genes (genotype 1, Table 2), which would constitute the fifth most common “species” in our study. These isolates had a similarity of 99.8% with *M. colombiense/intracellulare/bouchedurhonense* (MAC members) based on the 16S rDNA and of 100% with a sequence previously classified as belonging to MAC based on the *rpoB* gene (19). However, the closest match to their *hsp65* short sequence was *M. scrofulaceum* (97.4% of similarity), while the highest similarity with a MAC member *hsp65* sequence was only 95.7%, below the 97.3% threshold previously suggested for MAC members for this gene (33). This result highlights the importance of using multiple genetic markers to achieve a comprehensive identification of the species involved (28, 43). Further studies would be required to conclude on the nature of these isolates.

Our results demonstrate that multiple NTM species could be associated with the occurrence of non-specific reactions in the skin test in cattle in Spain. Nevertheless, the relative importance of MAC members (especially *Mah* and *Maa*) and *M. nonchromogenicum*, which constituted up to 69% of all isolates considered in the study, demonstrates that certain species are more likely to lead to diagnostic interferences in OTF herds in low prevalence/OTF areas of the country, in agreement with what has been described in other European (6, 15, 17–19) and non-European countries (34, 38, 39).

By including only isolates retrieved from positive animals in OTF herds (that maintained this status for at least 3 years after the reactor was found) we attempted to minimize the chance that animals could have also been infected with a MTC member. Further studies based on guinea pig and cattle experimental models will be conducted to confirm the ability of the characterized strains to induce non-specific reactions in non-bTB infected cattle, and to explore the ability of different antigens to discriminate the immune response induced in skin test and interferon-gamma assays. Altogether, this will contribute to limit the interference in the results of routine diagnostic tests, which can compromise the reliability of the herd status obtained and the non-desirable risk management measures that are necessary to implement in the context of an eradication programs.

## Data availability statement

The datasets presented in this study can be found in online repositories. The names of the repository/repositories and accession number(s) can be found below: https://www.ncbi.nlm.nih.gov/genbank/, OR642807—OR642833, OR648408—OR648468, OR671981—OR672046.

## Ethics statement

Ethical approval was not required for the study involving animals in accordance with the local legislation and institutional requirements because no need. All the animals were slaughtered due to positive results to the official skin test following the Bovine Tuberculosis Eradication Program in Spain.

## Author contributions

AG-B: Data curation, Formal analysis, Writing—original draft, Writing—review & editing. JAl: Data curation, Funding acquisition, Resources, Supervision, Writing—review & editing. JB: Funding acquisition, Writing—review & editing. JM: Conceptualization, Supervision, Writing—review & editing. JAm: Data curation, Resources, Writing—review & editing. JS: Investigation, Resources, Writing—review & editing. LdJ: Data curation, Funding acquisition, Writing—review & editing. BR: Data curation, Methodology, Supervision, Writing—review & editing.
