# Supplementary material for: Non-tuberculous mycobacteria: occurrence in skin test cattle reactors from official tuberculosis-free herds
Source: Front Vet Sci. 2024 Jan 31;11:1361788. doi: 10.3389/fvets.2024.1361788 (PMC10864654; doi:10.3389/fvets.2024.1361788)
Supplement: Supplementary file 1 [file Table_1.DOCX]

Supplementary Material

Non-Tuberculous Mycobacteria: Occurrence in Skin Test Cattle Reactors from Official Tuberculosis-Free Herds

Alberto Gomez-Buendia^1,2^, Julio Alvarez^1,2^, Javier Bezos^1,2^, Jorge Moruelo^3^, Javier Amado^4^, José Luis Saez^5^, Lucia de Juan^1,2^ and Beatriz Romero^1,2*^

*** Correspondence:** Beatriz Romero: bromerom@visavet.ucm.es

**Supplementary Table 1.** Primers used for the mycobacterial identification.

| Target DNA | Primers | Sequence 5’- 3’ | Size (bp) | Reference |
| --- | --- | --- | --- | --- |
| *mpb70* | TB1 F  TB1 R | *AGA GTT TGA TCC TGG CTC AG*  *AGC ACG CTG TCA ATC ATG TA* | 372 | (1) |
| 16S rRNA | Mycgen F  Mycgen R | *AGA GTT TGA TCC TGG CTC AG*  *TGC ACA CAG GCC ACA AGG GA* | 1030 | (1) |
| 16S rRNA | Mycgen F  Mav | *AGA GTT TGA TCC TGG CTC AG*  *ACC AGA AGA CAT GCG TCT TG* | 180 | (1) |
| IS*901* | IS901 F  IS901 R | *GCA ACG GTT GTT GCT TGA AA*  *TGA TAC GGC CGG AAT CGC GT* | 1108 | (2) |
| IS*1245* | IS1245 F  IS1245 R | *GCC GCC GAA ACG ATC TAC*  *AGG TGG CGT CGA GGA AGA* | 427 | (3) |
| *hsp65* short | TB11  TB12 | *ACC AAC GAT GGT GTG TCC AT*  *CTT GTC GAA CCG CAT ACC CT* | 441 | (4,5) |
| *rpoΒ* | Myco F  Myco R | GGC AAG GTC ACC CCG AAG GG  AGC GGC TGC TGG GTG ATC ATC | 711 | (6) |
| *hsp65* long | MAChspF 574  MAChsp65R | CGG TTC GAC AAG GGT TAC AT  ACG GAC TCA GAA GTC CAT GC | 1059 | (7) |
| 1. Wilton S, Cousins D. Detection and identification of multiple mycobacterial pathogens by DNA amplification in a single tube. Genome Res. 1992 May;1(4):269–73.  2. Kunze ZM, Portaels F, McFadden JJ. Biologically distinct subtypes of Mycobacterium avium differ in possession of insertion sequence IS901. J Clin Microbiol. 1992 Sep;30(9):2366–72.  3. Guerrero C, Bernasconi C, Burki D, Bodmer T, Telenti A. A novel insertion element from *Mycobacterium avium*, IS1245, is a specific target for analysis of strain relatedness. J Clin Microbiol. 1995 Feb;33(2):304–7.  4. Swanson DS, Kapur V, Stockbauer K, Pan X, Fronthingham R, Musser JM. Subspecific Differentiation of Mycobacterium avium Complex Strains by Automated Sequencing of a Region of the Gene (hsp65) Encoding a 65-Kilodalton Heat Shock Protein. Int J Syst Bacteriol. 1997 Apr 1;47(2):414–9.  5. Telenti A, Marchesi F, Balz M, Bally F, Böttger EC, Bodmer T. Rapid identification of mycobacteria to the species level by polymerase chain reaction and restriction enzyme analysis. J Clin Microbiol [Internet]. 1993 Feb;31(2):175–8. Available from: https://journals.asm.org/doi/10.1128/jcm.31.2.175-178.1993  6. Adékambi T, Drancourt M. Dissection of phylogenetic relationships among 19 rapidly growing *Mycobacterium* species by 16S rRNA, hsp65, sodA, recA and rpoB gene sequencing. Int J Syst Evol Microbiol. 2004 Nov;54(6):2095–105.  7. Turenne CY, Semret M, Cousins D V., Collins DM, Behr MA. Sequencing of hsp65 distinguishes among subsets of the *Mycobacterium avium* complex. J Clin Microbiol. 2006 Feb;44(2):433–40. | | | | |
